# Knowledge, attitudes, and practices about influenza illness and vaccination: a cross‐sectional survey in two South African communities

**DOI:** 10.1111/irv.12388

**Published:** 2016-04-29

**Authors:** Karen K. Wong, Adam L. Cohen, Shane A. Norris, Neil A. Martinson, Claire von Mollendorf, Stefano Tempia, Sibongile Walaza, Shabir A. Madhi, Meredith L. McMorrow, Ebrahim Variava, Katlego M. Motlhaoleng, Cheryl Cohen

**Affiliations:** ^1^ Centers for Disease ControlAtlantaGAUSA; ^2^ United States Public Health ServiceAtlantaGAUSA; ^3^ University of WitwatersrandJohannesburgSouth Africa; ^4^ MRC Developmental Pathways for Health Research UnitUniversity of WitwatersrandJohannesburgSouth Africa; ^5^ Johns Hopkins UniversityBaltimoreMDUSA; ^6^ National Institute for Communicable DiseasesJohannesburgSouth Africa; ^7^ Klerksdorp‐Tshepong Hospital ComplexKlerksdorpSouth Africa

**Keywords:** Influenza, South Africa, survey, vaccination

## Abstract

**Background:**

Understanding knowledge and sentiment toward influenza and vaccination is important for effective health messages and prevention strategies. We aimed to characterize knowledge, attitudes, and practices surrounding influenza illness and vaccination in two South African communities and explore reasons for vaccine hesitancy.

**Methods:**

Household primary caregivers in Soweto and Klerksdorp townships were interviewed about knowledge of influenza and intention to receive an influenza vaccine using a structured questionnaire. Factors associated with unwillingness to receive vaccine were explored using multivariable regression.

**Results:**

We interviewed representatives of 973 households in Soweto and 1,442 in Klerksdorp. Most respondents in Soweto (692, 71%) and Klerksdorp (1247, 87%) thought weather or cold caused influenza. While most would get a free influenza vaccine, those unwilling to receive vaccine had concerns about efficacy (Soweto: 19%; Klerksdorp: 19%) and safety (Soweto: 17%; Klerksdorp: 10%). In Soweto, females (aRR 2·0, 95% CI 1·3–3·2) and those with higher household income (aRR 1·8, 95% CI 1·2–2·7) were less willing to receive vaccine. In Klerksdorp, more educated respondents (aRR 1·6, 95% CI 1·1–2·4) were less willing to receive vaccine; households reporting an HIV‐positive member were more willing to receive vaccine (aRR 0·3, 95% CI 0·1–0·8).

**Conclusions:**

Although findings suggest most community participants were amenable to influenza vaccination, knowledge gaps were present. Emphasizing the importance of influenza as a health problem and addressing vaccine safety and efficacy concerns may improve uptake. Populations less amenable to vaccination, including those with higher education and income, may benefit from targeted messaging efforts.

## Background

Influenza is an important cause of morbidity and mortality globally and in South Africa. It is estimated that more than 450 children <5 years old and 9000 individuals ≥5 years old die each year from influenza in South Africa.^1^, ^2^ Influenza infections are responsible for 43–67% of outpatient visits for influenza‐like illness during the peak of influenza season.^3^ Among South Africans of 65 years of age or older, the rate of excess mortality due to pneumonia and influenza is estimated at 340 deaths per 100 000 population;^4^ this influenza‐attributable mortality rate is even higher among young adults with AIDS in South Africa, at an estimated 570 deaths per 100 000.^5^ In addition, HIV‐positive South Africans have been shown to be at greater risk for severe influenza illness.^6^ The high prevalence of underlying high‐risk medical conditions, including HIV and tuberculosis, likely contributes to higher influenza‐associated mortality in South Africa.^7^, ^8^

Vaccination and antiviral treatment may mitigate the impact of influenza in South Africa. Annual vaccination for seasonal influenza has been recommended in South Africa for certain high‐risk groups: young children, the elderly, pregnant women, and individuals with underlying medical conditions such as HIV.^9^ Influenza vaccines and antiviral medications are available in South Africa,^10^ although vaccine coverage remains low.^9^, ^11^ Understanding the knowledge, attitudes, and practices surrounding influenza and vaccination is important to improve the uptake of influenza prevention strategies in these communities.

The knowledge and beliefs surrounding influenza illness and vaccination among community members in South Africa have not previously been reported. In this study, we aim to characterize the knowledge, attitudes, and practices surrounding influenza illness and vaccination in one urban and one peri‐urban South African community.

## Methods

### Study setting

The study was conducted from August through September 2012, near the end of a typical influenza season,^3^ in two South African sites. Soweto, an urban township near Johannesburg in Gauteng Province, has an population of approximately 1·3 million people; Klerksdorp, in North West Province, includes peri‐urban gold mining townships Jouberton, Alabama, Sakhrol, Kanana, Khuma, Tigane, Dominionville, and Vaal Reefs, which have a combined population of over 274 000.^12^

### Study design and household selection

We conducted a cross‐sectional survey as part of a larger assessment of healthcare utilization for common infectious syndromes. Briefly, households were selected for participation by a simple random sample of geographic coordinates (latitude and longitude) within the boundaries of what were considered the residential areas of each site. The closest dwelling within 30 m of the randomly selected coordinates was approached for enrollment.

### Data collection

A team of two or three trained interviewers visited each household up to three times on separate days, including evenings and Saturdays as needed, to interview the primary caregiver in each household, defined as the person who self‐identified as knowing most about the health of the household members. Interviews were conducted in the preferred language of the caregiver (English, Xhosa, Setswana, or Zulu). The wording in the different languages was refined during prior discussions with the multilingual interviewers to ensure consistent interpretation of the questions and responses. The primary caregiver was asked to provide information on behalf of the entire household. Interviewers were instructed to read questions about influenza and vaccines without providing a list of possible answers, then to record the responses according to pre‐determined categories. If the response did not correspond to a pre‐determined category, the interviewer was instructed to mark “Other” and record the response as free text. Willingness to receive influenza vaccine was assessed by asking, “If it were available for free, would you get a vaccine against influenza?” Primary caregivers who answered “no” were then asked, “Why would you not get the vaccine?” One interviewer led the questionnaire, and the other members of the team would cross‐check the responses; any uncertainty in how to categorize responses was noted and resolved with the study team afterward.

### Ethical considerations

The purpose of the study was explained to the primary caregiver, who provided written consent for participation. Respondents were informed that they could choose to participate in the study, they could choose which questions to answer, they could stop the interview at any time, and the answers collected would be kept private to the extent allowed by law. The study was determined to be within the scope of public health practice by the US Centers for Disease Control and Prevention (ID #2012 6165), and it received approval by the University of Witwatersrand Human Research Ethics Committee (Medical) (Approval #M120367) and local authority approval from North West Province and Matlosana Subdistrict.

### Statistical analysis

Analysis was conducted using sas 9·3 (Cary, NC, USA). To compare two or more proportions, the chi‐square test or the Fisher's exact test was used, as appropriate. *P* < 0·05 was considered statistically significant. The Wilcoxon rank sum test was used to evaluate the difference in medians between two samples. Variables associated with unwillingness to receive vaccine with a significance level of *P* < 0·2 in an unadjusted analysis were selected for inclusion in the multivariable log‐binomial regression model to examine associations between individual characteristics and unwillingness to receive vaccine. Relative risks and adjusted relative risks are reported.

## Results

### Participants

Of 1,713 coordinates visited in Soweto, 191 (11%) did not have a dwelling within 30 meters of the coordinate from which a household could be enrolled. Of the 1522 households remaining, 207 (14%) declined participation, 342 (22%) did not have a primary caregiver available for enrollment after three attempted visits. Therefore, 973 (64%) completed the questionnaire. In Klerksdorp, 1,669 coordinates were visited and 57 (3%) did not have a dwelling located within 30 meters of the coordinate; of the 1612 households remaining, 51 (3%) declined participation, 119 (7%) did not have a primary caregiver available for enrollment after three attempted visits; 1,442 (89%) completed the questionnaire.

### Descriptive data

More primary caregivers were female in Klerksdorp (1026, 71%) than in Soweto (648, 67%; *P* = 0·02; Table 1). The median age of primary caregivers in Soweto and Klerksdorp was 40 years (IQR: 29–54 years) and 38 years (IQR: 29–49 years), respectively (*P* < 0·001). Between the two sites, there was a similar proportion of households having at least one child younger than 5 years (Soweto: 339, 35%; Klerksdorp: 538, 37%; *P* = 0·2), and a similar proportion of households with at least one pregnant woman (Soweto: 43, 4%; Klerksdorp: 62, 4%; *P* = 0·9). There was a higher proportion of households in Soweto (189, 19%) compared with Klerksdorp (205, 14%) that had at least one household member ≥65 years old (*P* < 0·001). A greater proportion of households in Klerksdorp (249, 17%) compared with Soweto (87, 9%) reported having at least one HIV‐positive household member (*P* < 0·0001).

**Table 1:** Characteristics of primary caregivers and their households—Soweto (*n* = 973) and Klerksdorp (*n* = 1442), South Africa, 2012

Characteristic	Soweto *n* (%)	Klerksdorp *n* (%)	*P*‐value
Female d	648 (67)	1026 (71)	.02
Age, years a ^,^ d			
18–34	586 (41)	360 (37)	.03
35–64	751 (52)	513 (53)	
≥65	104 (7)	97 (10)	
Household monthly income b ^,^ d			
<R500 (<59 USD)	91 (11)	128 (10)	<.0001
R500–1000 (59–118 USD)	162 (20)	236 (18)	
R1000–2000 (118–237 USD)	285 (36)	536 (41)	
R2000–5000 (237–592 USD)	150 (19)	241 (18)	
R5000–10 000 (592–1183 USD)	67 (8)	134 (10)	
R10 000–15 000 (1183–1775 USD)	22 (3)	21 (2)	
> R15 0000 (>1775 USD)	25 (3)	8 (1)	
Highest level of education completed c ^,^ d			
No school	31 (3)	102 (7)	<.0001
Some primary	65 (7)	222 (15)	
Completed primary	43 (4)	118 (8)	
Some secondary	389 (40)	545 (38)	
Grade 12/matriculation	313 (33)	411 (29)	
College/University	121 (13)	35 (2)	
Trade school	0	1 (<1)	
Number of people in household d			
1	69 (7)	120 (8)	.01
2	138 (14)	226 (16)	
3–5	528 (54)	757 (52)	
6–10	206 (21)	320 (22)	
11+	32 (3)	19 (1)	
Child <5 years old in household	339 (35)	538 (37)	.2
HIV‐infected person in household d	87 (9)	249 (17)	<.0001
Pregnant woman in household	43 (4)	62 (4)	.9
Person age ≥65 in household d	189 (19)	205 (14)	.0007

a Percents reported out of 1441 in Klerksdorp and 970 in Soweto.

b Percents reported out of 1304 in Klerksdorp and 802 in Soweto. ZAR to USD conversions as of September 1, 2012^13^.

c Percents reported out of 1434 in Klerksdorp and 962 in Soweto.

d Chi‐square test significant at alpha = 0·05.

### Influenza and vaccination knowledge

Most primary caregivers identified cough as a symptom of influenza (Soweto: 590, 61%; Klerksdorp: 970, 67%; Table 2). The majority of primary caregivers in Soweto (692, 71%) and Klerksdorp (1247, 87%) thought that weather or cold caused influenza; there were 123 (13%) in Soweto and 118 (8%) in Klerksdorp who said they did not know what caused influenza. There were 204 (21%) primary caregivers in Soweto and 437 (30%) in Klerksdorp who did not believe people could die from influenza.

**Table 2:** Knowledge and attitudes regarding influenza and sources of health information—Soweto (*n* = 973) and Klerksdorp (*n* = 1442), South Africa, 2012

Survey question	Soweto *n* (%)	Klerksdorp *n* (%)	*P*‐value
What symptoms are associated with influenza? a			
Cough	590 (61)	970 (67)	0·0008
Runny nose, stuffy nose, sneezing	513 (53)	801 (56)	0·2
Fever, chills, sweats	387 (40)	647 (45)	0·01
Headache	142 (15)	151 (10)	0·002
Sore throat	95 (10)	92 (6)	0·002
Fatigue	64 (7)	37 (3)	<0·0001
Diarrhea, nausea, vomiting, abdominal pain	61 (6)	41 (3)	<0·0001
Other symptoms	130 (13)	106 (7)	<0·0001
Don't know	41 (4)	20 (1)	<0·001
What causes influenza? b			
Weather or cold	692 (71)	1247 (87)	<0·0001
Virus, bacteria, or germs	204 (21)	76 (5)	<0·0001
Dirty environment	107 (11)	76 (5)	<0·0001
Other	72 (7)	16 (1)	<0·0001
Don't know	123 (13)	118 (8)	0·0003
Can people die from influenza? c			
Yes	642 (66)	855 (59)	<0·0001
No	204 (21)	437 (30)	
Don't know	125 (13)	147 (10)	
Where do you get most of your information on health‐related matters? d			
Clinics or hospitals	497 (51)	909 (63)	<0·0001
Television	187 (19)	168 (12)	
Radio	124 (13)	291 (20)	
Newspapers	61 (6)	24 (2)	
Internet	19 (2)	7 (<1)	
Friends or relatives	15 (2)	21 (1)	
Church or religious organization	7 (1)	4 (<1)	
Traditional healers	0	0	
Other	53 (5)	10 (1)	
Don't know	9 (1)	8 (1)	

a Percents reported out of 1441 in Klerksdorp and 973 in Soweto. More than one response could be selected.

b Percents reported out of 1440 in Klerksdorp and 972 in Soweto. More than one response could be selected.

c Percents reported out of 1439 in Klerksdorp and 971 in Soweto.

d Percents reported out of 1442 in Klerksdorp and 972 in Soweto.

While most primary caregivers in Soweto (645, 66%) and Klerksdorp (836, 58%) reported that the purpose of a vaccine is to prevent disease, there were 215 (22%) in Soweto and 559 (39%) in Klerksdorp who did not know the purpose of a vaccine (Table 3). Over half of primary caregivers in Soweto (602, 63%) and Klerksdorp (822, 57%) believed that a vaccine could protect people from influenza. In Soweto, 764 (79%) primary caregivers said they would get an influenza vaccine if it were free; in Klerksdorp, this proportion (1326, 92%) was significantly higher (*P* < 0·001).

**Table 3:** Knowledge, attitudes, and practices regarding influenza vaccination—Soweto (*n* = 973) and Klerksdorp (*n* = 1442), South Africa, 2012

Survey question	Soweto *n* (%)	Klerksdorp *n* (%)	*P*‐value
What is the purpose of a vaccine? a			
Prevent disease	645 (66)	836 (58)	<0·0001
Make disease less severe	39 (4)	23 (2)	0·0002
Treat or cure disease	52 (5)	15 (1)	<0·0001
Other purpose	20 (2)	6 (<1)	0·0001
Don't know	215 (22)	559 (39)	<0·0001
Do you think a vaccine can protect people from influenza? b			
Yes	602 (63)	822 (57)	<0·0001
No	110 (11)	42 (3)	
Don't know	245 (26)	572 (40)	
If it were available for free, would you get a vaccine against influenza? c			
Yes	764 (79)	1326 (92)	<0·0001
No	119 (12)	91 (6)	
Don't know	87 (9)	20 (1)	
Reasons that primary caregiver would not want a free vaccine against influenza d			
Vaccine would not work	23 (19)	17 (19)	0·9
Vaccine is not safe	20 (17)	9 (10)	0·2
I do not know enough about it	10 (8)	9 (10)	0·7
Injections are painful	7 (6)	11 (12)	0·1
Vaccine is not important	3 (3)	7 (8)	0·08
Needles are not safe	3 (3)	5 (5)	0·3
Influenza is not dangerous	1 (1)	2 (2)	0·4
I do not need it/I am not sick	5 (4)	9 (10)	0·1
Other/Don't know	25 (3)	10 (1)	0·0002
Would you want anyone in your household to get a vaccine against influenza? e			
Yes	798 (83)	1341 (93)	<0·0001
No	79 (8)	65 (5)	
Don't know	86 (9)	31 (2)	
Where would you go first if you wanted to get the flu vaccine for yourself or someone in your household? f			
Clinic	731 (76)	1215 (85)	<0·0001
Public hospital	47 (5)	74 (5)	
Private hospital	70 (7)	46 (3)	
Pharmacy	47 (5)	49 (3)	
Would not get vaccine	11 (1)	33 (2)	
Don't know	52 (5)	14 (1)	

a Percents reported out of 1442 in Klerksdorp and 972 in Soweto. More than one response could be selected.

b Percents reported out of 1436 in Klerksdorp and 957 in Soweto.

c Percents reported out of 1437 in Klerksdorp and 970 in Soweto.

d Percents reported out of 91 in Klerksdorp and 119 in Soweto. More than one response could be selected.

e Percents reported out of 1437 in Klerksdorp and 963 in Soweto.

f Percents reported out of 1431 in Klerksdorp and 958 in Soweto.

### Factors associated with unwillingness to receive vaccine

The most common reason people were unwilling to receive an influenza vaccine was that they believed that the vaccine would not prevent influenza (Soweto: 23, 19%; Klerksdorp: 17, 19%; *P* = 0·9; Table 3). Concerns about vaccine safety were more prevalent in Soweto (20, 17%) than in Klerksdorp (9, 10%), although this difference was not statistically significant (*P* = 0·2). There were 10 (8%) primary caregivers in Soweto and 9 (10%) in Klerksdorp who said they would not want a vaccine because they do not know enough about it.

Among participants in Soweto, unwillingness to receive vaccine was adjusted for primary caregiver age ≥35 years, primary caregiver sex, monthly household income of ≥ZAR 2000 (≥ 237 USD, as of September 1, 2012),^13^ education level of completing at least Grade 12, and a household member ≥ 65 years old, <5 years old, with self‐reported HIV‐positive status, or a pregnant household member. After adjusting for these characteristics, unwillingness to receive vaccine in Soweto remained significantly associated with a female primary caregiver (aRR 2·0, 95% CI 1·3–3·2) and higher household monthly income (aRR 1·8, 95% CI 1·2–2·7) (Table 4). In Klerksdorp, the multivariable model was adjusted for primary caregiver sex, education level, and having self‐reported HIV‐positive or pregnant household members. Households in Klerksdorp were significantly less willing to receive vaccine if the primary caregiver had a higher education level (aRR 1·6, 95% CI 1·1–2·4), and they were significantly more willing to receive vaccine if a household member was reported to be HIV‐positive (aRR 0·3, 95% CI 0·1–0·8).

**Table 4:** Factors associated with unwillingness to receive influenza vaccine^c^—Soweto and Klerksdorp, South Africa, 2012

Characteristic	Soweto			Klerksdorp		
	Would not want vaccine, *n*/*N* (%)	RR (95% CI)	aRR (95% CI)	Would not want vaccine, n/N (%)	RR (95% CI)	aRR (95% CI)
Respondent age ≥35y	88/610 (14)	1·7 (1·1–2·5)^a^	1·5 (0·9–2·3)	53/855 (6)	1·0 (0·6–1·4)	b
Respondent age <35y	31/363 (9)	Ref	Ref	38/587 (6)		
Female respondent	92/648 (14)	1·7 (1·1–2·6)^a^	2·0 (1·3–3·2)^a^	55/1026 (5)	0·6 (0·4–0·9)^a^	0·7 (0·5–1·1)
Male respondent	27/325 (8)	Ref	Ref	36/416 (9)		
Higher income^d^	47/264 (18)	1·9 (1·3–2·7)^a^	1·8 (1·2–2·7)^a^	27/404 (7)	1·3 (0·8–2·1)	b
Lower income	51/538 (9)	Ref	Ref	46/900 (5)		
Higher education^e^	64/434 (15)	1·5 (1·1–2·1)^a^	1·3 (0·9–2·0)	40/447 (9)	1·8 (1·2–2·6)^a^	1·6 (1·1–2·4)^a^
Lower education	52/528 (10)	Ref	Ref	50/987 (5)		
Person ≥65y in household	32/189 (17)	1·5 (1·1–2·2)^a^	1·4 (0·9–2·1)	10/205 (5)	0·7 (0·4–1·4)	b
No person ≥65y in household	87/784 (11)	Ref	Ref	81/1237 (7)		
Child <5y in household	29/339 (9)	0·6 (0·4–0·9)^a^	0·6 (0·4–1·0)^a^	33/538 (6)	1·0 (0·6–1·4)	b
No child <5y in household	90/634 (14)	Ref	Ref	58/904 (6)		
HIV‐infected person in household^f^	6/87 (7)	0·5 (0·2–1·2)	0·6 (0·3–1·4)	5/249 (2)	0·3 (0·1–0·7)^a^	0·3 (0·1–0·8)^a^
No HIV‐infected person in household	113/886 (13)	Ref	Ref	86/1193 (7)		
Pregnant woman in household	2/43 (5)	0·4 (0·1–1·4)	0·3 (0·05–2·4)	1/62 (2)	0·2 (0·03–1·7)	0·3 (0·04–1·9)
No pregnant woman in household	117/930 (13)	Ref	Ref	90/1380 (7)		

RR, relative risk; aRR, adjusted relative risk; CI: confidence interval; ref: referent value.

a *P* < 0·05.

b Not included in multivariable model.

c Answered “no” to question, “If it were available for free, would you get a vaccine against influenza?”

d Household monthly income ≥ R2000 versus < R2000.

e Highest education level of respondent Grade 12 or higher versus less than Grade 12.

f Self‐reported HIV status.

Among those who said they would not want to receive an influenza vaccine, there were 104 (Soweto: 64; Klerksdorp: 40) primary caregivers who reported a higher education level (≥Grade 12) and 102 (Soweto: 52; Klerksdorp: 50) who reported a lower education level (<Grade 12). Both high and low education level groups reported some similar reasons for unwillingness to receive vaccine, including believing that the vaccine would not work (high education: 21, 20%; low education: 19, 19%), concerns that vaccine is not safe (high education: 13, 13%; low education: 16, 16%), and not knowing enough about the vaccine (high education: 12, 12%; low education, 7, 7%). Thirteen primary caregivers with higher education levels stated other reasons for being unwilling to receive vaccine, of which 8 (62%) were related to non‐specific personal preferences such as “no specific reason,” “don't like it,” or “hospital is not my thing.” In contrast, only 1 (6%) of 16 primary caregivers in the lower education group stated a non‐specific preference (“I don't like injections”) (*P* = 0·004). The other primary caregivers with lower education level cited specific reasons, such as “once tried it and was sick for 3 months”, “I believe in God and prayer,” “believe in natural remedies,” “I know how to treat influenza,” “it's done yearly,” and “don't want to get used to it.” Among those with higher education level, specific reasons cited for unwillingness to receive vaccine included “allergic to injections,” “free things don't guarantee,” and “it has drugs and it's natural to have flu.”

## Discussion

Although some knowledge gaps and misconceptions about influenza illness and vaccination were identified in these communities, most participants reported a willingness to receive an influenza vaccine. Among those who indicated that they would not want vaccination, common reasons for unwillingness included concerns about vaccine efficacy and safety. We found that those with higher education levels or household incomes were less willing to receive vaccination. However, households that include members of certain high‐risk groups, such as those with HIV‐positive household members, may be more amenable to influenza vaccination.

### Knowledge gaps about influenza and vaccination

We identified knowledge gaps among primary caregivers about influenza illness and vaccination. The description of symptoms suggests that influenza, or the colloquial term “flu,” is often thought of as a mild upper respiratory infection. Many respondents did not think that people could die from influenza. However, the rate of influenza‐attributable death among older adults in South Africa is several times higher than it is among older adults in the United States.^4^ This underestimation of influenza severity in the community may undermine its importance as a public health issue. The tendency to dismiss influenza, especially compared to competing public health issues such as HIV and tuberculosis, is a barrier to improving influenza prevention and treatment strategies.^14^ Influenza was often reported to be caused by cold or weather rather than by a virus; addressing this misconception in the community may improve adherence to non‐pharmaceutical interventions for reducing influenza transmission, such as hand hygiene and avoiding contact with sick persons.

There was limited understanding of the purpose of a vaccine, and many people did not think a vaccine could protect people against influenza. A study of South African youth similarly found low levels of understanding of the purpose of vaccination.^15^ Despite limited vaccine knowledge, most primary caregivers said they would get an influenza vaccine if available for free, although this proportion varied by site. While studies of vaccine acceptability among primary caretakers in the community are limited, willingness to accept influenza vaccine in this study was similar to acceptability observed among healthcare workers in Kenya^16^ and Cote d'Ivoire^17^ and higher than acceptability observed among pregnant women in Cote d'Ivoire^18^ during the 2009 influenza A (H1N1) pandemic. We did not explore how clinician recommendations, which have been shown to influence vaccination attitudes and practices,^19^ might affect willingness to receive influenza vaccine in this population. Health messaging in South African communities should consider highlighting influenza as a disease distinct from upper respiratory infections and should clarify that influenza can cause severe outcomes that may be mitigated by vaccination and other prevention strategies.

### Factors associated with unwillingness to receive vaccine

Some primary caregivers said they would not want a free influenza vaccine, and exploring their reasons for unwillingness to receive vaccine is critical to addressing prevention strategies in these communities. The most common reason was belief that the vaccine would not work. Addressing vaccine effectiveness in health messages may improve willingness to be vaccinated in these communities. Some participants stated they would not want vaccination because they held beliefs in complementary therapies or religion, implying that they perceived these to be exclusive of allopathic therapies such as vaccination. This perception, which has been noted previously in South Africa,^20^ may be important for planning health messages that accepting vaccination does not preclude complementary belief systems.

Those with higher income and education were less willing to receive vaccine. Previous studies that examined factors associated with vaccine uptake found both positive and negative associations with education level and income.^21^, ^22^, ^23^, ^24^ In this study, we found that many reasons for unwillingness to receive vaccine were similar in higher and lower education groups; however, the qualitative responses of some participants may suggest that while those with lower education levels have specific beliefs or reasons for not wanting vaccine, those with higher education levels have non‐specific preferences not to get vaccinated.

In Soweto, female respondents were less willing to receive an influenza vaccine than male respondents; however, this difference was not observed in Klerksdorp. Both communities demonstrated an association between unwillingness to receive vaccine and having a household member in a high‐risk group, although the high‐risk group differed by site (child <5 years old in Soweto, HIV‐infected person in Klerksdorp). There may be is a common perception that certain people are at higher risk for influenza complications and therefore would benefit from vaccination; however, the beliefs about which groups are at higher risk may be specific to each community. Understanding the regional differences in factors associated with vaccination attitudes is important to develop effective and targeted health messaging.

### Health belief model

The findings can be interpreted within the Health Belief Model, a framework for relating factors that lead to change in health‐related behavior.^25^ Framing the severity of influenza as a health threat to individuals and household members who may be at higher risk, or relating the illness to lost wages due to missing work, may help communities understand the negative consequences in influenza infection. This study suggests that those with lower education levels who were unwilling to receive vaccine cited specific reasons for their choice; health messaging for this group that addresses some of the specific benefit‐versus‐cost concerns and emphasizes the importance of influenza as health problem may influence attitudes about vaccination. Among those with higher education levels who cited more non‐specific reasons for being unwilling to receive vaccination, clarifying the underlying concerns may enable more effective health messaging. Most participants believed in their own ability to obtain a vaccine if they wanted one.

### Limitations

This study is subject to certain limitations. Several households were not able to be surveyed because the primary caregiver was unavailable. The primary caregiver of the household was asked to respond on behalf of the household, and demographic characteristics of these respondents do not represent those of the general community. Nonetheless, surveying the primary caregiver is useful as this is often the person making health decisions of other household members. Characteristics of other household members, such as age, pregnancy status, and HIV status, were reported by the primary caregiver or by the household member if present, and may be subject to misclassification error; conditions such as HIV are likely to be underreported. HIV prevalence reported in this study is much lower than reported by other surveys of the general population;^26^ it is likely that underreporting of HIV status would make it more difficult to detect an association between HIV status and vaccine attitudes. It is unclear whether underreporting of health conditions may be positively or negatively associated with influenza vaccine sentiment. This survey was only implemented in one urban site and one peri‐urban site, and results may not be generalizable to other South African communities. In this survey, reasons for being unwilling to receive vaccination were examined, but reasons for wanting influenza vaccination, including clinician recommendations, were not explored. Factors that improve acceptance of vaccination are important to understand to design effective public health interventions. Finally, we are assessing hypothetical acceptability of influenza vaccine, when faced with a real decision, choices may differ.

## Conclusions

Despite these limitations, this survey was able to identify knowledge gaps about influenza illness and vaccination and describe potential barriers, based on these misconceptions, to influenza prevention strategies. Emphasizing the potential severity of influenza and its yearly morbidity and mortality can improve awareness of influenza as a public health problem. Targeting influenza illness and vaccine messages to address the specific concerns uncovered in this study, especially among groups less amenable to vaccination, may improve effectiveness of influenza prevention strategies.

## Disclaimer

The findings and conclusions in this study are those of the authors and do not necessarily represent the views of the Centers for Disease Control and Prevention.

## Addendum

KKW, CvM, NM, SN, ST, SW, SM, MM, EV, CC, and AC conceptualized the study. KKW, CvM, NM, SN, ST, SW, KMM, CC, and AC implemented the field study. KKW performed the statistical analysis and drafted the initial manuscript. All authors interpreted the data, reviewed and revised the manuscript, and approved the final manuscript.

## Competing interests

The authors declare that they have no competing interests.
